# Census of Health: Tentative Suggestions

**Published:** 1920-02-28

**Authors:** 


### THE PUBLIC WELL-BEING.

### Census of Health: Tentative Suggestions.

When the next Census is taken—the Minister of Health says it will be early next year—there will be a good opportunity for the Ministry of Health, which will presumably conduct the Census, to get more detailed records of the state of national health. Statistics of the physical condition of the male adult population were comprehensively obtained under the Military Service Acts, and though so definite a toll cannot very well be collected in a census of the whole population of all sexes and ages made without the possibility of universal medical examination, there might be fuller information than that provided in the 1915 Census, which, on the score of well-being, only required answers to the question whether any person was blind, deaf, dumb, imbecile or lunatic. It required, also, a tabulation of the number of children born and the number living. The former of these two categories might easily be extended, and the latter (regarding children) might well be taken a little further. Even if no additional information were asked for, the statistics of the male population already obtained would make next year's Census the most complete account yet taken.

There is some point, however, in the suggestion of the committee appointed by the Royal Statistical Society, that results obtained from questions on infirmities have been misleading and inaccurate in the past. But in some form or other, a health Census, if it could be reasonably complete, would be of enormous value. Diseases compulsorily notifiable and death statistics are the only strictly universal data which the authorities now get from the whole population. The school children statistics are most important, but they are limited to certain sections of the community. Local medical officers of health can do much in these directions.

Following a payment scheme either in compensation for the professional time involved or for each individual recorded, would there be objections to all medical practitioners making returns, without any disclosure of individuals, as to the categories and statistics of diseases within their practices?

